# Non-Echocardiographic Evaluation of Hypertrophic Cardiomyopathy

**DOI:** 10.31083/RCM51298

**Published:** 2026-10-12

**Authors:** Karim Ali, Ahmed E. Ali, Mohamed K. Awad, Kyle Wansing, Mohamad Elgozair, Mohamed Abdalla, Cooper Gearhart, Ahmed Sami Abuzaid

**Affiliations:** ^1^Department of Internal Medicine, Hennepin County Medical Center, Minneapolis, MN 55415, USA; ^2^Department of Internal Medicine, Crestwood Medical Center, Huntsville, AL 35801, USA; ^3^Department of Critical Care, Faculty of Medicine, Ain Shams University, 1181 Cairo, Egypt; ^4^Department of Internal Medicine, Zucker School of Medicine at Hofstra/Northwell, Danbury, CT 06810, USA; ^5^Neuroscience Department, Case Western Reserve University, Cleveland, OH 44106, USA; ^6^Alaska Heart and Vascular Institute, University of Alaska, Anchorage, AK 99571, USA

**Keywords:** hypertrophic cardiomyopathy, diagnostic imaging, magnetic resonance imaging, cardiac, electrocardiography, radionuclide imaging, myocardial fibrosis, risk assessment, phenotype

## Abstract

Hypertrophic cardiomyopathy (HCM) is a heterogeneous genetic and phenotypic myocardial disease where diagnosis and risk stratification extend beyond the mere detection of left ventricular hypertrophy. While echocardiography is the first-line imaging modality, this approach often offers limited insights into disease etiology, biological activity, and natural history. Thus, several non-echocardiographic modalities have emerged as prominent components of contemporary HCM investigations. Electrocardiography (ECG) provides early, readily accessible insights into myocardial remodeling and arrhythmic risk. Cardiac magnetic resonance (CMR) imaging enhances phenotyping through detailed tissue characterization and fibrosis assessment, while nuclear imaging uniquely evaluates myocardial perfusion and microvascular injury. Plasma biomarkers and genetic testing offer valuable insights into disease activity and risk stratification for affected individuals and at-risk relatives, especially when combined with imaging findings. Therefore, this review summarizes current knowledge on non-echocardiographic approaches to HCM, highlighting the associated complementary strengths and the value of integrated multimodality assessment in improving disease phenotyping, prognostication, and management. Importantly, integrating these modalities improves risk stratification for sudden cardiac death, heart failure progression, and arrhythmias, guiding decisions on implantable cardioverter-defibrillator (ICD) placement and medical management. This approach supports more personalized, mechanism-based care in HCM.

## 1. Introduction

Hypertrophic cardiomyopathy (HCM) remains an area of ongoing clinical challenge in cardiovascular medicine. Many patients with the genetic substrate for disease are asymptomatic, but some become progressively symptomatic, develop atrial and ventricular arrhythmias, or experience sudden death, occasionally at a young age [1]. The broad range of clinical phenotypes reflects the interplay of genetic substrate, myocardial remodeling, and modifying clinical factors, and it limits the usefulness of any single diagnostic test [2].

In recent years, the clinical landscape of HCM has evolved significantly, with improved risk stratification and widespread use of implantable cardioverter-defibrillators contributing to a decline in sudden cardiac death rates. Consequently, heart failure progression, driven by diastolic dysfunction, microvascular ischemia, and myocardial fibrosis, has emerged as a major determinant of morbidity and mortality. This shift underscores the need for diagnostic strategies that extend beyond structural assessment to capture disease activity, progression, and individualized risk [1,2].

Echocardiography remains a cornerstone for identifying left ventricular hypertrophy and dynamic obstruction but hypertrophy itself is a relatively uninformative finding when it comes to the etiology, stage, or familial risk of the disease [3]. Wall thickening can be caused by many different molecular and metabolic processes, and genotype-positive disease may exist in the absence of overt structural changes. Echocardiography alone may therefore be insufficient to answer the crucial questions regarding the prognostic implications of disease or familial risk [4].

Non-echocardiographic testing provides complementary answers to address these questions. Fig. 1 summarizes the key takeaway points from this review. Fig. 2 illustrates a practical multimodality workflow for HCM evaluation. Initial assessment typically begins with echocardiography, ECG, and biomarkers to establish the presence of hypertrophy, obstruction, electrical remodeling, or myocardial stress. These findings then guide second-line investigations such as CMR for fibrosis characterization and risk stratification, cardiac computed tomography (CT) when anatomical clarification is needed, and nuclear imaging for assessment of coronary microvascular dysfunction. Genetic testing and artificial intelligence (AI)-enabled multimodal integration further refine diagnosis, familial evaluation, and individualized prognostic assessment. Importantly, these modalities should be viewed as complementary rather than competing tools within a stepwise clinical framework [3,4]. 

**Fig. 1.**
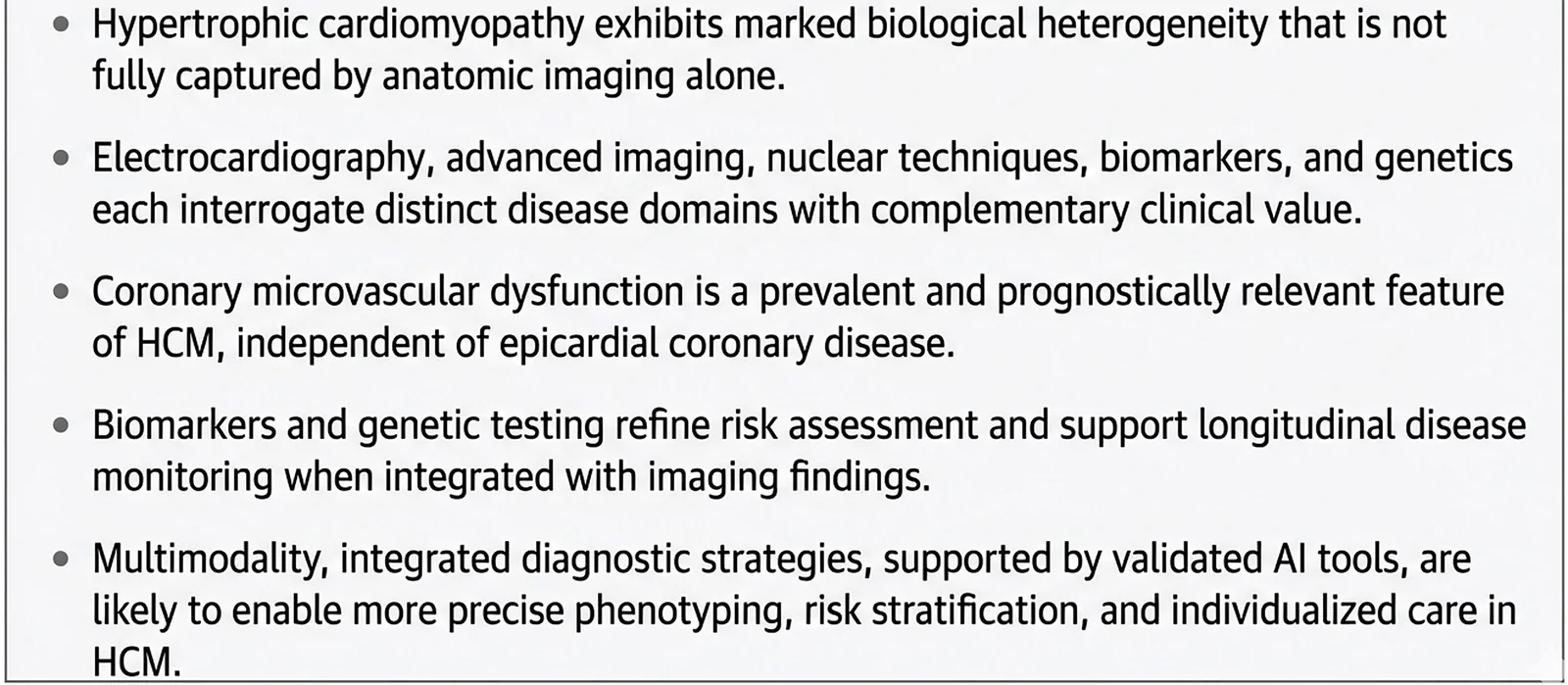
**Five takeaway points**. HCM, hypertrophic cardiomyopathy; AI, artificial intelligence. Created in BioRender.

**Fig. 2.**
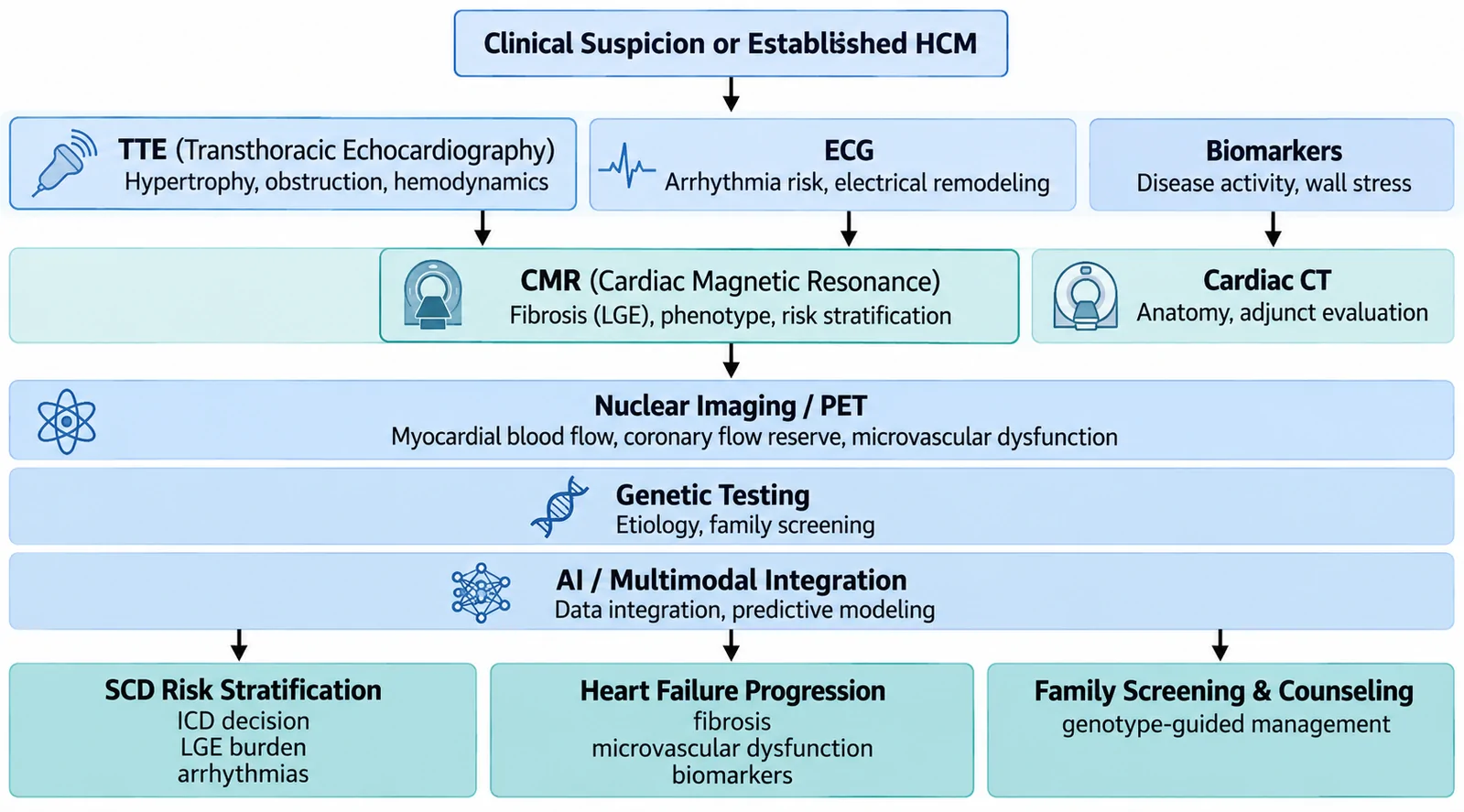
**Multimodality evaluation of HCM: integrated diagnostic and management workflow**. ECG, electrocardiography; CT, computed tomography; PET, positron emission tomography; SCD, sudden cardiac death; ICD, implantable cardioverter-defibrillator; LGE, late gadolinium enhancement. Created in BioRender.

## 2. Electrocardiography (ECG)

ECG remains a cornerstone in the evaluation of HCM given its universal availability, low cost, and ability to reflect both myocardial architecture and electrical remodeling (Fig. 3). Current guidelines recommend a standard 12-lead ECG at initial evaluation and every one to two years during longitudinal follow-up [5].

**Fig. 3.**
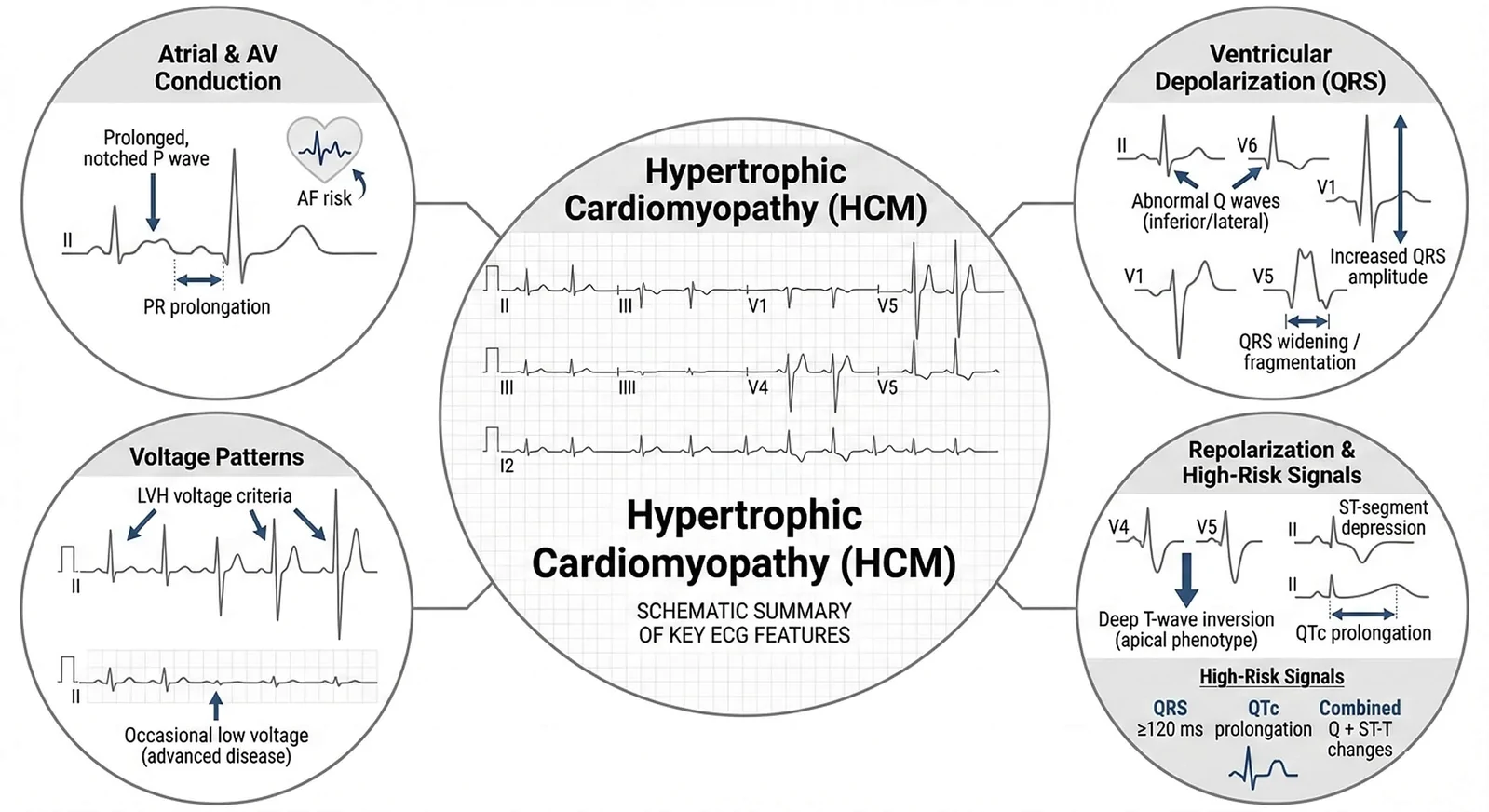
**Electrocardiographic hallmarks of hypertrophic cardiomyopathy**. AV, atrioventricular; AF, atrial fibrillation; QTc, corrected QT interval. Created in BioRender.

ECG abnormalities are detected in approximately 75–95% of phenotypically expressed HCM patients [5]. A completely normal ECG is uncommon and generally confined to early or mild disease, including some genotype-positive, phenotype-negative carriers [6]. No single ECG finding is pathognomonic for HCM; diagnostic value emerges from recognizing characteristic patterns in clinical and imaging context.

### 2.1 Atrial and Atrioventricular Conduction Abnormalities

Left atrial abnormality, manifested by P-wave prolongation (>120 ms), a broad, bifid, or notched P wave in lead II and V1, or a prominent terminal negative deflection in V1, is among the most frequent ECG findings and reflects chronically elevated filling pressures and diastolic dysfunction. P-wave duration is the strongest independent ECG predictor of atrial fibrillation (AF) in HCM, which occurs in 20–25% of patients [7]. It’s associated with a 7-fold increased thromboembolic risk, a 2.8-fold increased risk of heart failure, and a 2.5-fold increased risk of all-cause mortality [8]. Stroke risk approximates 3% per year regardless of conventional risk scores, and anticoagulation, preferably with direct oral anticoagulants, is recommended after the first AF episode without reliance on CHA_2_DS_2_-VASc scoring, which is not informative in HCM [9]. Therefore, Identification of P-wave abnormalities should prompt closer rhythm surveillance with ambulatory monitoring and early discussion of anticoagulation.

PR interval prolongation (≥200 ms), which is present in ~23% of patients, correlates with greater septal thickness, larger left atrial size, elevated filling pressures, and more extensive fibrosis. It has been associated with increased all-cause mortality in some cohorts [10]. Early-onset or rapidly progressive atrioventricular (AV) block is atypical for sarcomeric HCM and should raise suspicion for phenocopies such as storage disorders or cardiac amyloidosis [11]. Conversely, a short PR with delta waves should prompt consideration of glycogen storage disease rather than sarcomeric HCM [12].

### 2.2 Ventricular Depolarization Abnormalities

Abnormal Q waves (amplitude ≥0.3 mV, depth ≥25% of the R wave, or duration >40 ms) are present in ~50% of patients, most commonly in inferior and lateral leads, and may mimic prior myocardial infarction [13]. When combined with repolarization changes (the QST pattern), specificity for sarcomeric mutation carriage is high and may precede overt hypertrophy [14].

Voltage criteria for left ventricular hypertrophy (LVH) are insensitive in HCM; only ~one-third of adolescents with phenotypic HCM meet conventional thresholds [15]. Combining Cornell with Lewis or Gubner-Ungerleider criteria improves sensitivity to ~76–80% while preserving specificity [16]. Importantly, low QRS voltage in a patient with apparent LVH should raise suspicion for a phenocopy, particularly cardiac amyloidosis, which classically produces a discordance between marked wall thickening on imaging and unexpectedly low ECG voltages. This pattern is diagnostically distinct from sarcomeric HCM, where low voltage reflects advanced fibrosis in a minority and portends a worse prognosis [17].

QRS duration ≥120 ms, present in ~17% of patients, independently predicts sudden cardiac death and major cardiovascular events [18]. Complete bundle branch block is relatively uncommon in HCM and is often secondary to septal myomectomy. In post-myomectomy patients, left bundle branch block occurs in 30–40% without affecting survival, while complete heart block requiring permanent pacemaker implantation occurs in <1% [19].

### 2.3 Repolarization Abnormalities

T-wave inversion ≥0.1 mV or ST depression ≥0.1 mV in inferior or lateral leads are highly prevalent in HCM. Deep precordial T-wave inversion is a hallmark of apical HCM and may be present before wall thickness thresholds are met [20]. Corrected QT interval (QTc) prolongation (mean 440–488 ms) is common; QTc ≥460 ms independently predicts ventricular arrhythmias and sudden cardiac death [21]. ST-segment elevation with negative T waves in advanced disease may signal apical aneurysm formation.

### 2.4 Prognostic Role of Non-Sustained Ventricular Tachycardia

Non-sustained ventricular tachycardia (NSVT) on ambulatory monitoring, defined as ≥3 consecutive ventricular beats at ≥120 bpm, is detected in 20–30% of HCM and carries dual prognostic significance. It’s an independent risk factor for sudden cardiac death and is incorporated into current sudden cardiac death (SCD) risk stratification models [5]. NSVT is also a marker of progressive heart failure, particularly when frequent or prolonged, and should prompt comprehensive risk reassessment including CMR for fibrosis quantification [5]. Guidelines recommend ambulatory monitoring to detect NSVT at initial evaluation and every 1–2 years thereafter, with implantable cardioverter-defibrillator (ICD) implantation considered when NSVT clusters with other major risk factors such as unexplained syncope, massive LVH, family history of sudden death, or apical aneurysm [5,10].

### 2.5 ECG Changes with Myosin Inhibitors

Mavacamten and aficamten, cardiac myosin inhibitors now used for obstructive HCM, produce characteristic ECG changes that clinicians should recognize [22]. Treatment is associated with modest QTc prolongation, a known dose-dependent effect of mavacamten that necessitates baseline ECG assessment and periodic monitoring during dose titration [22]. Reductions in LVH voltage and improvement in repolarization abnormalities have also been observed with effective myosin inhibition, likely reflecting reduced dynamic obstruction and wall stress [23]. ECG monitoring is therefore both a safety requirement and a potential marker of therapeutic response in patients receiving these agents.

### 2.6 Temporal Evolution

ECG abnormalities in HCM are dynamic. Early disease may manifest rising QRS voltages and emerging T-wave changes. Over subsequent decades, progressive left atrial enlargement and QRS prolongation reflect worsening diastolic dysfunction and conduction system involvement. Advanced disease may feature left bundle branch block and attenuated QRS voltages as fibrosis predominates.

## 3. Cardiac Computed Tomography (CT)

### 3.1 Established Applications of Cardiac CT in Cardiomyopathies

The use of CT in the evaluation of cardiomyopathies is increasing, despite not being a first-line imaging modality [24]. Echocardiography is widely accessible and routinely performed, but its diagnostic utility may be limited by poor acoustic windows in some patients, and it has been shown to be less sensitive than CMR for detecting hypertrophic cardiomyopathy in certain cases [25]. CMR avoids the image-quality limitations and provides excellent tissue characterization, but its use can be restricted in many centers due to limited availability, the need for specialized expertise, and competition for scanner time with other clinical services [26]. A further limitation is that a subset of patients are unable to tolerate the procedure because of claustrophobia [27]. In addition, the presence of pacemakers or implantable cardioverter-defibrillators is frequently viewed as a contraindication, even though established protocols have demonstrated that CMR can be performed safely in these patients [28,29]. In this context, current guidelines support the use of cardiac CT as a complementary imaging modality in patients with inconclusive echocardiography or when CMR is contraindicated or not feasible [30].

Cardiac CT offers several advantages over CMR in the assessment of HCM. It is widely available and does not require beta-blockade unless coronary imaging is needed. CT allows extremely rapid data acquisition, with comprehensive protocols completed in approximately seven minutes [26]. Advances in rapid gantry rotation, now fast enough in some systems to capture the beating heart within a single cardiac cycle and without requiring table motion, combined with sub-millimeter isotropic volume elements that provide fine 3D detail, have led to remarkably high-quality cardiac CT imaging [31].

Cardiac CT provides precise delineation of the myocardium and allows accurate measurement of cardiac wall thickness, ventricular volumes, left ventricular mass, and ejection fraction, all of which show good correlation with CMR findings. In addition, cardiac computed tomography (CCT) enables evaluation of coronary arteries and cardiac valves [32].

Coronary assessment is an important established application of coronary computed tomography angiography (CCTA) in hypertrophic cardiomyopathy. CCTA can noninvasively detect atherosclerotic plaques, characterize stenosis, and quantify plaque burden, providing high-resolution anatomical detail of the coronary arteries [33]. In symptomatic HCM patients, approximately 20% have been found to have clinically significant atherosclerosis [34]. While obstructive coronary artery disease may contribute to angina in this population, its prevalence, severity, and plaque characteristics vary across studies [33]. Notably, some evidence suggests that the increased coronary luminal volume commonly seen in HCM may allow plaques to be accommodated without producing significant stenosis [35], highlighting both the diagnostic and prognostic value of CCTA in assessing coronary anatomy in these patients.

In the setting of myocardial bridging (MB), CCTA allows detailed anatomical visualization of the coronary artery lumen and surrounding myocardium. Muscle bridging can be classified as partial or complete, depending on the extent to which the coronary segment is surrounded by myocardium. CCTA provides noninvasive, high-resolution imaging and is particularly useful in adults with HCM for detecting MB [36,37].

Beyond diagnosis and morphological assessment, CT plays an important role in planning septal reduction therapies for HCM [38]. It can precisely map the septal perforator arteries and their relationship to the obstructing septal hypertrophy (Fig. 4), helping determine whether a patient is an appropriate candidate for alcohol septal ablation, which is strongly influenced by perforator anatomy [39]. CT can also be used to assess infarct size after ablation with good agreement compared to CMR, supporting its value in treatment planning and follow up [40].

**Fig. 4.**
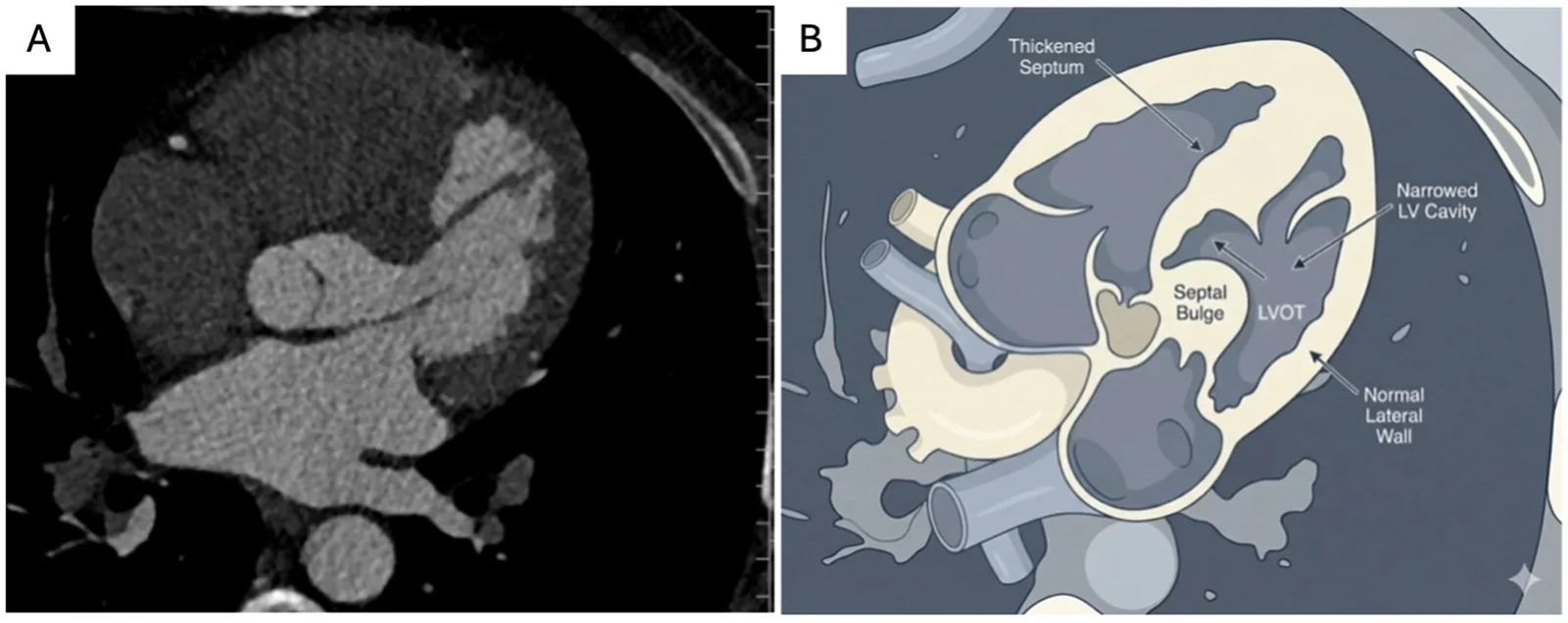
**Asymmetric septal hypertrophy on cardiac computed tomography**. (A) shows a contrast-enhanced long-axis cardiac CT demonstrating asymmetric septal hypertrophy. (B) provides a schematic illustration highlighting key anatomic features relevant to phenotyping and septal reduction planning. LV, left ventricle; LVOT, left ventricular outflow tract; CT, computed tomography. Created in BioRender.

### 3.2 Investigational and Emerging Applications

Beyond simple wall thickening, CCT can reveal more subtle morphological features often seen in patients with hypertrophic cardiomyopathy. These include quantitative left ventricle (LV) strain assessment using CT feature tracking [41], myocardial crypts [42], anomalous muscle bundles [43], apical displacement of the papillary muscles [44], increased fractal complexity of the LV endocardial surface [45] and early detection of small apical aneurysms, which may be difficult to identify on echocardiography but have important prognostic implications [26].

In HCM, coronary microvascular dysfunction is frequently observed and is associated with increased myocardial wall thickness as well as the presence of late gadolinium enhancement (LGE) on CMR, reflecting myocardial fibrosis [46]. CT myocardial perfusion imaging provides a noninvasive method to evaluate myocardial blood flow by tracking the distribution of contrast during its first pass through the myocardium, which is directly dependent on the coronary arterial supply [47]. In single-energy CT, early arterial phase images (8–16 seconds) reveal areas of reduced attenuation corresponding to impaired perfusion or ischemia, typically acquired at rest and after pharmacologic stress to detect reversible defects [48]. Dual-energy CT generates iodine-based perfusion maps, enabling quantification of myocardial blood flow and precise identification of perfusion abnormalities, supporting clinical assessment of coronary artery disease [49]. However, these techniques are still under investigation and not yet widely adopted in routine clinical practice.

Similarly, delayed enhancement CT detects myocardial fibrosis in high-risk HCM patients, identifying areas that may serve as a substrate for life-threatening arrhythmias and aiding risk stratification, with studies showing good correlation between delayed myocardial enhancement by CT and by CMR [50].

Moreover, 4D reconstructions make it possible to examine the intricate relationships between the interventricular septum, papillary muscles, and mitral valve, providing insights that remain challenging to obtain with cine CMR and would otherwise require invasive transesophageal echocardiography [26]. The clinical impact of these techniques, however, continues to be evaluated.

### 3.3 Limitations of Cardiac CT

The administration of a contrast agent carries notable risks, including allergic reactions, hyperthyroidism, and worsening of renal function. Contrast induced nephropathy (CIN) is particularly concerning, and numerous studies have shown a dose dependent relationship between the volume of contrast administered and the likelihood of renal impairment [51]. Cardiac CT protocols often use contrast volumes up to 150 mL, which, although higher than in many standard coronary CT scans and improving image quality, also may increases the potential risk of CIN [52].

Another major limitation of cardiac CT is radiation exposure. This can be minimized using strategies such as prospective ECG triggered acquisition, high pitch helical scanning, modulation of tube current based on the ECG, adjustment of tube current according to body region or patient habitus, lowering kVp and mAs, using thicker slices, and applying iterative reconstruction techniques to preserve image quality at reduced doses [24]. Additionally, cardiac CT has suboptimal performance in patients with poorly controlled atrial fibrillation, as arrhythmia can degrade image quality and limit diagnostic accuracy [26].

## 4. Cardiac Magnetic Resonance Imaging (CMR)

### 4.1 Pathophysiology and Imaging Targets

HCM encompasses a broad spectrum of CMR phenotypes and imaging features, as illustrated in Fig. 5. To understand the importance of CMR, it is helpful to link imaging findings to the underlying HCM pathophysiology. The disease is characterized by cardiomyocyte hypertrophy, myocyte disarray, and interstitial fibrosis, which lead to asymmetric wall thickening, reduced ventricular compliance, and dynamic left ventricular outflow tract (LVOT) obstruction.

**Fig. 5.**
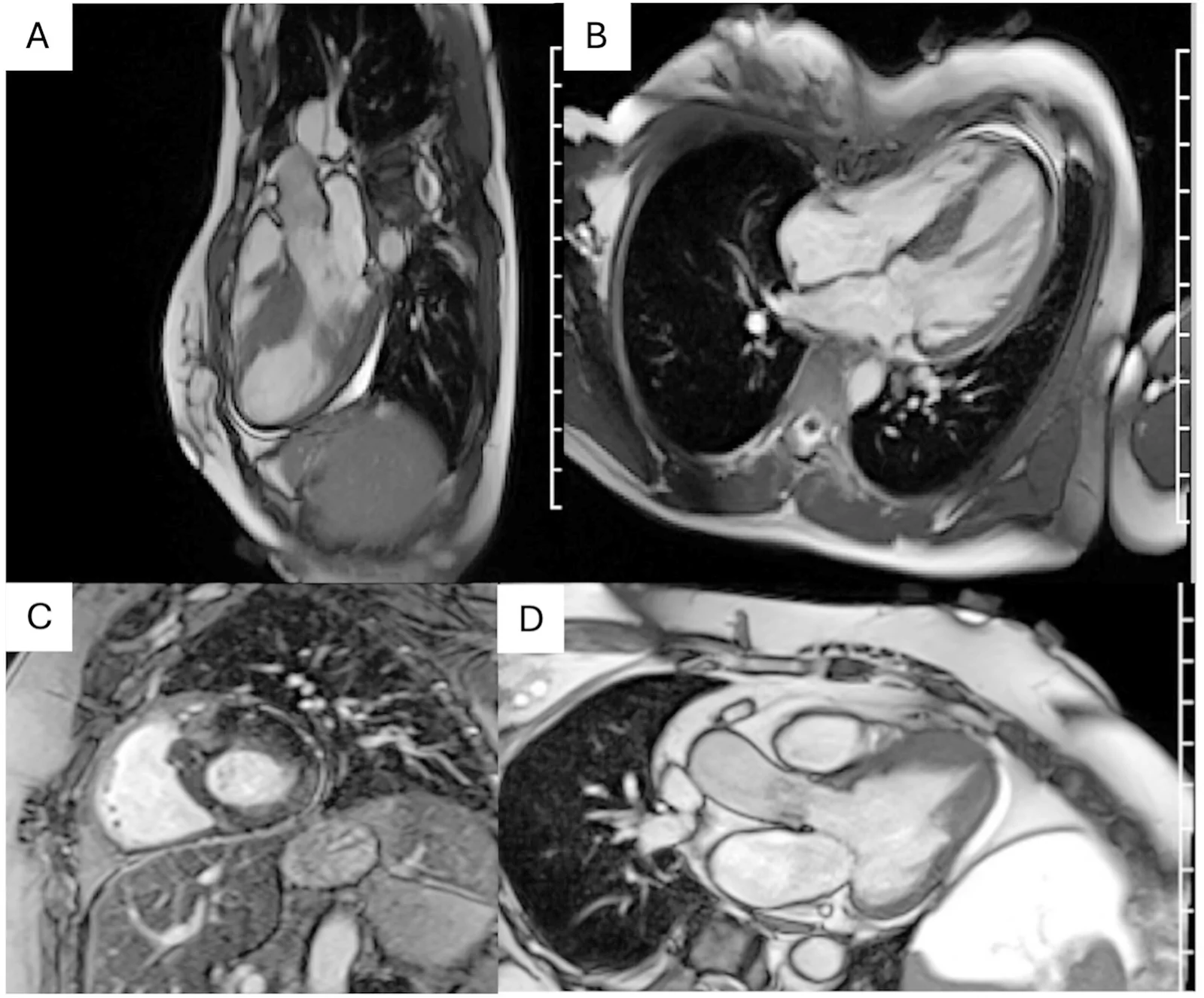
**Cardiac magnetic resonance (CMR) phenotypes in hypertrophic cardiomyopathy (HCM)**. (A,B) show steady-state free precession cine images demonstrating left ventricular hypertrophy. (A) (three-chamber view) shows maximal basal septal hypertrophy measuring 1.5 cm. (B) (four-chamber view) demonstrates milder basal inferoseptal hypertrophy (1.3 cm) with a prominent apical–basal muscle bundle contributing to obstructive physiology. (C) shows a short-axis PSIR (phase-sensitive inversion recovery) image demonstrating septal hypertrophy with late gadolinium enhancement at the right ventricular insertion points. (D) shows a long-axis steady-state free precession (SSFP) cine image demonstrating apical hypertrophy without evidence of apical aneurysm.

### 4.2 Myocardial Hypertrophy and Remodeling

The hallmark of HCM is hypertrophy, but its distribution is often heterogeneous. The basal interventricular septum remains the archetypal site (Fig. 5A,B), yet hypertrophy may be confined to the apex (Fig. 5C,D), involve the mid-ventricle, or extend to the right ventricle, patterns that can be easily underappreciated on echocardiography. CMR, with its high spatial resolution and full ventricular view, delineates these morphologic variants and allows precise quantification of maximal wall thickness (MWT), a key input in contemporary sudden cardiac death risk models [53,54].

Remodeling in HCM is not limited to the myocardium but also involves the mitral valve apparatus. Elongation of the anterior mitral leaflet and abnormal papillary muscle morphology or insertion frequently contributes to LVOT obstruction. CMR offers comprehensive visualization of these valvular and subvalvular structures, helping to distinguish fixed from dynamic components of obstruction and to inform management strategies [53,54].

### 4.3 Myocardial Fibrosis

Fibrosis in HCM can occur in two forms, both can be assessed by CMR (Fig. 5C): Replacement Fibrosis: This represents focal macroscopic scarring that often results from microvascular ischemia and myocyte death. It can be visualized using LGE sequences and is typically found in the hypertrophied segments and at the insertion points of the right ventricle into the septum [55].

Interstitial Fibrosis: This is a diffuse expansion of the extracellular matrix due to collagen deposition between myocytes. It can be detected using T1 mapping and extracellular volume (ECV) quantification. This diffuse process can precede focal scarring and occurs independently of regional hypertrophy [56].

### 4.4 Microvascular Dysfunction

Coronary microvascular dysfunction (CMD) is a core feature of HCM, driven by medial hypertrophy of intramural arterioles and extrinsic compression by hypertrophied myocardium. This supply-to-demand mismatch leads to ischemia that induces fibrosis and arrhythmias, and can be identified by vasodilator stress perfusion CMR, which frequently shows severe subendocardial ischemia despite nonobstructive epicardial coronary arteries [57,58].

### 4.5 Myocyte Disarray

Myocyte disarray is the histologic hallmark of HCM but was historically confined to autopsy and surgical specimens. Diffusion Tensor Imaging (DTI) now allows for the *in vivo* assessment of myocardial sheet function and fiber orientation, providing a direct insight into the primary disease histology [59].

### 4.6 CMR Acquisition Protocols and Methodology

The society for cardiovascular magnetic resonance (SCMR) has established standardized protocols to ensure diagnostic consistency and reproducibility across centers. A comprehensive HCM protocol is multiparametric, integrating anatomy, function, tissue characterization, and hemodynamics [5,60].

### 4.7 Cine Imaging: The Foundation of Morphology

Steady-state free precession (SSFP) cine imaging in standard long-axis views and a contiguous short-axis stack remains the backbone of CMR in HCM because it provides the most accurate assessment of wall thickness, chamber volumes, ejection fraction, and mitral/subvalvular anatomy [53]. These measurements help in confirming the diagnosis, recognition of atypical phenotypes, planning of therapy, and sudden cardiac death risk assessment.

### 4.8 Late Gadolinium Enhancement (LGE): Visualizing the Scar

LGE is central to scar imaging in HCM and is most clinically relevant for identifying replacement fibrosis and quantifying fibrosis burden for risk stratification (Fig. 5C). In clinical practice, the key value of LGE is identifying the presence, distribution, and extent of scar, because these features inform diagnosis, arrhythmic risk assessment, and longitudinal follow-up [61].

### 4.9 Parametric Mapping: Quantifying the Substrate

Parametric mapping provides absolute, quantitative tissue characterization and is particularly useful when disease is diffuse and conventional LGE may underestimate burden because no truly normal myocardial reference is available [56,62].

Native T1 Mapping: Native T1 is typically elevated in HCM and helps distinguish HCM from phenocopies such as Fabry disease, athlete’s heart, and hypertensive remodeling [62,63]. Beyond diagnosis, higher native T1 appears to reflect more advanced diffuse disease and should be viewed as a marker of disease severity that complements other risk markers.

ECV Fraction: ECV quantifies interstitial expansion and often detects diffuse fibrosis in segments without visible LGE. In HCM, elevated ECV supports pathological rather than physiological hypertrophy and has prognostic relevance, particularly for heart-failure progression and other adverse outcomes related to diffuse myocardial remodeling [64].

T2 Mapping: T2 mapping is sensitive to myocardial water content and can identify edema or active myocardial injury. In HCM, T2 elevation likely reflects an active disease component driven by microvascular ischemia, supply-demand mismatch, myocyte injury, and superimposed inflammatory or immune activation rather than fibrosis alone [65,66]. Studies linking high T2 signal or elevated T2 values to troponin release, ventricular arrhythmias, and worse outcomes support this interpretation [67]. Accordingly, abnormal T2 should be interpreted as a marker of ongoing injury that may refine risk assessment, particularly when present alongside LGE and conventional clinical markers.

### 4.10 Diffusion Tensor Imaging (DTI)

DTI is an emerging technique that provides insight into myocardial microstructure. Although reduced fractional anisotropy (FA) and altered diffusion metrics may detect disarray before overt hypertrophy as shown in genotype-positive/phenotype-negative (G+P-) carriers, limited availability, sequence complexity, and lack of standardized clinical thresholds currently preclude routine use in everyday HCM management [59,68,69].

### 4.11 4D Flow MR

4D flow CMR can visualize complex LVOT and aortic flow in HCM and quantify parameters such as peak gradients, wall shear stress, and viscous energy loss. Its current value is mainly investigational; it may deepen understanding of obstruction and adverse remodeling, but it is not yet part of routine risk stratification or everyday decision-making [70,71].

### 4.12 Phenotypic Heterogeneity and Diagnosis

The diagnosis of HCM is clinical and imaging-based, defined by an MWT ≥15 mm in adults, or 13–14 mm in the presence of a family history or pathogenic sarcomeric variant, after excluding secondary causes of hypertrophy. CMR is particularly useful for defining the full spectrum of morphologic phenotypes that may be underestimated or missed on echocardiography [5].

### 4.13 Asymmetric Septal Hypertrophy (ASH)

This is the classic and most common phenotype, characterized by disproportionate thickening of the interventricular septum relative to the posterior wall (typically defined as a septal-to-posterior wall thickness ratio >1.3 in normotensive patients). The hypertrophy typically involves the basal and mid-septum. CMR can accurately define the longitudinal extent of hypertrophy, distinguishing between focal basal involvement and extensive diffuse thickening [53].

### 4.14 Apical HCM (Yamaguchi Syndrome)

Apical HCM is characterized by hypertrophy confined to the LV apex, creating a spade-like cavity in diastole (Fig. 5D). This variant is frequently underdiagnosed on transthoracic echocardiography (TTE) due to near-field artifacts, foreshortening of the apex, and sometimes, poor acoustic windows [60]. CMR can reliably visualize the obliteration of the apical cavity and detect the presence of small apical aneurysms, which are often obscured on echo and carry a high risk of thrombus formation and stroke [72].

### 4.15 Mid-Ventricular Obstruction

In this variant, hypertrophy is maximal at the mid-ventricular level, creating an hourglass configuration and a mid‑cavity pressure gradient. This phenotype is strongly associated with the development of apical aneurysms due to chronic high pressure in the apical chamber [73].

### 4.16 Mass-Negative (G+P-) Phenotypes

Genetic testing has identified a cohort of patients who carry pathogenic sarcomere mutations but do not manifest LVH yet; the G+P- group. CMR can reveal subtle pre‑hypertrophic changes that support closer follow‑up. Myocardial Crypts which are deep, blood-filled invaginations within the myocardium, most commonly in the basal inferoseptal wall, are found in >60% of G+P- carriers but are rare in healthy controls or those with overt HCM [43]. Other features, such as anterior mitral leaflet elongation and increased trabecular complexity with mildly elevated ECV, may also indicate early disease expression before overt wall thickening develops [54].

### 4.17 The Grey Zone of Hypertrophy

Patients with mild LV wall thickening in the 13–15 mm range fall into a diagnostic “grey zone”, where HCM must be distinguished from hypertensive remodeling and athlete’s heart. In this setting, CMR contributes by integrating morphology, LGE, mapping indices, and additional features such as crypts or mitral valve abnormalities to support or refute a diagnosis of early HCM.

### 4.18 Differential Diagnosis: Distinguishing Phenocopies

One of the most powerful applications of CMR is its ability to distinguish HCM from physiological adaptations and infiltrative storage diseases (phenocopies). This differentiation is critical as treatments and prognoses are totally different. Summary of the differentiating features for HCM and other phenocopies is summarized in Table 1.

**Table 1.** **Differentiating HCM from phenocopies using CMR**.

Feature	Hypertrophic cardiomyopathy (HCM)	Hypertensive heart disease (HHD)	Athlete’s heart	Cardiac amyloidosis (CA)	Anderson-Fabry disease (AFD)
Morphology	Asymmetric septal hypertrophy (common), apical hypertrophy. MWT ≥15 mm.	Concentric (usually), MWT usually <15 mm.	Concentric. The hypertrophy is reversible and regresses with detraining.	Concentric thickening (including RV and atria).	Concentric (usually).
LGE Pattern	Presents with patchy, mid-wall fibrosis, particularly at right ventricular insertion points.	May show no enhancement or a non-specific patchy mid-wall pattern.	None (or minor insertion point).	Global subendocardial or transmural enhancement, with difficult nulling of the myocardium.	Mid-wall enhancement, especially in the basal inferolateral wall.
Native T1	High, reflecting diffuse myocardial fibrosis.	Normal or slightly elevated.	Normal or low, consistent with cellular hypertrophy.	Very high (>1100 ms at 1.5 T), indicating amyloid infiltration.	Low due to lipid storage within the myocardium.
ECV	Elevated (>25–28%).	Normal or slightly elevated.	Normal or low (<24%).	Very High (>40–50%).	Normal.
Strain (GLS)	Reduced (–14%), no specific pattern.	Mildly reduced or normal.	Normal.	Demonstrates apical sparing (“cherry on top” pattern).	Reduced in the basal inferolateral segments.
Other	Additional findings can include myocardial crypts and elongated mitral valve leaflets.	Frequently associated with a dilated left atrium.	Enlarged left and right ventricular volumes are typical.	May be accompanied by pericardial effusion and atrial septal thickening.	There may be T2 signal elevation in the basal inferolateral wall.

MWT, maximum wall thickness; RV, right ventricle; LGE, late gadolinium enhancement; T1, native T1 relaxation time (T1 mapping); ECV, extracellular volume fraction; GLS, global longitudinal strain; T2, T2 relaxation time (T2-weighted imaging / T2 mapping).

### 4.19 Risk Stratification and Prognosis

The major catastrophic risk in HCM is SCD from ventricular arrhythmias. Identifying patients who may benefit from ICD placement is therefore a central role of imaging. CMR adds prognostic information that complements the established clinical markers and guideline-based risk models. In practice, LGE burden, left ventricular ejection fraction (LVEF), maximal wall thickness, and apical aneurysm should be integrated with history, rhythm monitoring, and family history during shared decision-making regarding ICD therapy. Key CMR prognostic markers in HCM are summarized in Table 2.

**Table 2.** **Key CMR prognostic markers in HCM**.

Marker	Threshold/definition	Clinical implication (SCD risk)
LGE Extent	Extensive scar; often ≥15% of LV mass, but threshold depends on quantification method.	Risk modifier for ICD decision-making; should be integrated with clinical risk markers rather than used in isolation.
Max Wall Thickness	≥30 mm.	High Risk. Major conventional risk factor.
Apical Aneurysm	Discrete aneurysm, ideally characterized for size, scar, and thrombus.	Major risk modifier in AHA/ACC guidance; more limited stand-alone role in ESC-based algorithms.
LVEF	<50%.	End-stage HCM. High risk of SCD and HF death.
Native T1	Elevated global T1.	Tracks diffuse disease burden and heart-failure-related risk.
ECV	Elevated global ECV.	Marker of diffuse interstitial fibrosis and adverse remodeling, especially heart-failure progression.
LV Entropy	High heterogeneity.	Predictor of SCD and ventricular arrhythmias.
Myocardial Disarray	Low Fractional Anisotropy (DTI).	Correlates with ventricular arrhythmias.

LGE, late gadolinium enhancement; LV, left ventricle; SCD, sudden cardiac death; AHA, American Heart Association; ACC, American College of Cardiology; ESC, European Society of Cardiology; LVEF, left ventricular ejection fraction; HF, heart failure; T1, longitudinal relaxation time (T1 mapping); ECV, extracellular volume fraction; DTI, diffusion tensor imaging.

### 4.20 Late Gadolinium Enhancement (LGE)

LGE is a strong predictor of adverse outcomes in HCM, with extensive evidence showing associations with SCD, appropriate ICD therapy, ventricular arrhythmia, and all-cause mortality. Importantly, its prognostic value relates not only to its presence but also to the extent and progression over time.

In a systematic review and meta-analysis by Kamp et al. [74], the authors analyzed eight prospective studies including 3808 patients and found that LGE positivity was associated with significantly higher odds of SCD (OR 1.69), aborted SCD or appropriate ICD therapy (OR 3.27), and all-cause mortality (OR 2.1). The progression of LGE over time also carries prognostic significance. Aquaro et al. [75] found that LGE rate >0.07 g/month was the best independent predictor of hard events and allowed significant risk reclassification compared to single-timepoint LGE measurements (net reclassification improvement 0.49, *p* = 0.003). In a multicenter study of 1293 patients, LGE showed a continuous relationship with SCD risk; each 10% increase in LGE burden was associated with a 46% increase in SCD events, and LGE ≥15% of LV mass demonstrated an approximately 2-fold higher SCD risk among patients otherwise considered to be at lower risk [76]. More recently, a meta-analysis of 11 studies including 5550 patients confirmed that quantitative LGE extent improves SCD risk stratification across different quantification methods. When the commonly used 6-SD method was analyzed, an LGE burden of approximately 10% provided the optimal discrimination for SCD, although with modest sensitivity of 0.73 and specificity of 0.67 [77]. Accordingly, contemporary guidelines support incorporating CMR-derived fibrosis burden into shared decision-making when ICD candidacy remains uncertain; the 2024 American Heart Association (AHA)/American College of Cardiology (ACC) and 2023 European Society of Cardiology (ESC) guidelines identify extensive LGE (≥15%) as a factor that may influence prophylactic ICD decisions. Thus, LGE extent should be viewed as an adjunctive risk modifier that complements rather than replaces established clinical and imaging markers [5,78].

However, LGE quantification has not been standardized. Reported methods include manual segmentation, signal-threshold-versus-reference-mean approaches using thresholds from 2 to 6 standard deviations above remote myocardium, and full-width at half-maximum techniques [79]. In HCM, where fibrosis is often patchy and heterogeneous rather than sharply demarcated, these methods can produce materially different estimates of scar burden. Earlier methodological studies showed substantial variation in measured LGE volume depending on the technique used, and in A recent meta-analysis, most studies used 6-SD but reported prognostic cutoffs ranging from 5.3% to 20% of LV mass [77]. More recent reproducibility data suggest that 5-SD and 6-SD perform similarly in HCM, but no universal standard has yet been adopted [79]. Therefore, LGE burden should be interpreted in the context of the acquisition and post-processing method used, and serial comparison is most reliable when the same technique is applied consistently.

### 4.21 Left Ventricular Apical Aneurysm

Apical aneurysms are clinically important for two reasons: they are associated with significantly elevated SCD risk and a propensity for intraventricular thrombus formation [80]. In a cohort study, patients with HCM apical aneurysms ≥2 cm had a 5-year SCD event rate around 10%, compared with 2.9% for aneurysm size <2 cm [81]. Another study reported an SCD event rate of almost 5% per year in this population [82]. The hazard ratio for SCD increases by 1.69 per 1-cm increase in aneurysm size [81]. Thromboembolic risk is equally concerning as patients with apical aneurysms experienced stroke or apical thrombus formation at a rate of 2.9% per year, with aneurysm size ≥2 cm; a hazard ratio of 2.20 for these events [81]. CMR is superior to echocardiography for detecting small aneurysms and for defining associated transmural scar or intracavitary thrombus, while contrast echocardiography or cardiac CT may also be useful when apical visualization is limited. Importantly, the presence of an apical aneurysm is not, by itself, a universal indication for anticoagulation; rather, it should prompt careful surveillance and dedicated imaging to exclude thrombus, particularly when the aneurysm is large or akinetic/dyskinetic. The approach to primary-prevention ICD also differs across guidelines. The 2024 AHA/ACC guideline treats LV apical aneurysm as a major risk factor that may support ICD implantation even when conventional risk estimates are borderline, whereas ESC-based algorithms have assigned a more limited role to aneurysm presence and LV apical aneurysm was not incorporated into the ESC-based HCM Risk-SCD calculator [5]. Therefore, management should be individualized, incorporating aneurysm size, scar burden, documented arrhythmias, systolic function, and evidence of thrombus rather than aneurysm presence alone.

### 4.22 Systolic Dysfunction (Burned-Out Phase)

Progression to LV systolic dysfunction (LVEF <50%) marks an end‑stage or burned‑out phase of HCM, usually in the setting of extensive fibrosis and LV remodeling. This is associated with a very high risk of SCD and heart failure death, warranting ICD implantation and evaluation for transplant. CMR is the best monitoring tool for tracking subtle declines over time and linking systolic impairment to the underlying scar burden [5,83].

### 4.23 Emerging Prognostic Markers

Quantitative mapping indices are increasingly recognized as complementary prognostic markers. Elevated global native T1 and ECV, including in non‑LGE myocardium, are independent predictors of heart failure hospitalization and cardiac death, reflecting the impact of diffuse interstitial disease beyond focal scar [84,85]. This reflects the burden of diffuse disease beyond focal scars. Additionally, LV entropy, a texture analysis metric derived from T1 mapping or LGE images reflecting the tissue heterogeneity, can serve as an independent predictor of SCD and cardiovascular mortality [86]. Reduced global longitudinal strain (GLS) is associated with cardiac adverse outcomes. CMR feature-tracking strain shows good overall agreement with speckle-tracking echocardiography (STE) for GLS and circumferential strain, and CMR can offer better assessment of regional and global deformation [87]; however, its interpretation should be cautious because feature-tracking measurements are affected by significant intervendor variability, particularly for radial and regional strain, and serial follow-up is most reliable when the same post-processing software is used consistently [87,88].

### 4.24 Hemodynamic Assessment: 4D Flow and Physiology

While morphology is static, HCM physiology is dynamic. The obstruction in the LVOT varies with loading conditions.

### 4.25 4D Flow CMR

4D Flow CMR acquires velocity data in three spatial dimensions over time, creating a complete velocity vector field of the heart.

**Gradient Assessment:** 4D Flow CMR enables comprehensive assessment of dynamic LVOT obstruction in HCM through time-resolved three-dimensional velocity mapping, allowing retrospective gradient quantification and visualization of complex flow patterns. Unlike Doppler echocardiography, which requires precise real-time alignment, 4D Flow permits retrospective analysis from any desired plane, particularly valuable for the eccentric, turbulent jets characteristic of HCM, and provides additional hemodynamic insights including energy losses, vorticity, and wall shear stress [71,89]. Studies demonstrate good diagnostic accuracy (71–100% sensitivity, 75–94% specificity) compared to echocardiography, with strong correlations between flow derangement and both LVOT gradients and adverse myocardial remodeling [71].

**Viscous Energy Loss:** 4D Flow quantifies the energy lost due to turbulence. HCM patients demonstrate significantly elevated energy loss in the LVOT and ascending aorta compared to controls. This metric correlates with the degree of hypertrophy and ECV, providing a measure of hemodynamic inefficiency [71].

**Wall Shear Stress (WSS):** In HCM, abnormal left ventricular outflow tract geometry generates eccentric, asymmetric flow jets that create helical and vortical flow patterns in the ascending aorta [89,90]. These disturbed flow patterns produce areas of elevated and asymmetrically distributed wall shear stress on the aortic wall, particularly affecting areas like the left coronary sinus with increased backward flow [90]. The chronic exposure to abnormal wall shear stress is associated with progressive aortic dilation through mechanically mediated structural alterations in the vessel wall, independent of underlying connective tissue abnormalities [91].

### 4.26 CMR Assessment of Microvascular Ischemia

Stress-induced perfusion defects typically manifest as subendocardial and circumferential patterns that are most pronounced in hypertrophied segments [92,93]. These patterns reflect the underlying microvascular dysfunction that is highly prevalent in HCM, occurring in approximately 40–79% of patients [92,93]. The perfusion abnormalities correlate with the degree of hypertrophy, with stress myocardial blood flow (MBF) decreasing as wall thickness and left ventricular mass increase [93].

The prognostic value of reduced myocardial perfusion reserve index (MPRI) is supported by multiple lines of evidence. Quantitative perfusion measurements demonstrate significantly reduced stress MBF and myocardial perfusion reserve (MPR) in HCM patients compared to controls [93]. In HCM cohorts, stress MBF averages 1.63 ± 0.60 mL/g/min versus 2.30 ± 0.64 mL/g/min in controls, with MPR similarly reduced (2.21 ± 0.87 versus 2.90 ± 0.90). Reduced MBF and MPR measured by CMR perfusion mapping provide strong, independent predictors of adverse cardiovascular outcomes [94].

The temporal relationship between perfusion abnormalities and LGE is particularly noteworthy. Perfusion defects can occur even in apparently normal myocardial segments without hypertrophy or LGE, suggesting that microvascular dysfunction may represent an early disease marker that precedes the development of replacement fibrosis [93]. Segments with reduced MPRI show associations with interstitial fibrosis (elevated extracellular volume) and are linked to worse ventilatory efficiency on exercise testing [95].

## 5. Nuclear Imaging and Microvascular Dysfunction in HCM

Nuclear imaging offers a functional perspective on HCM that complements anatomic assessment by other diagnostic modalities including echocardiography, CT, and CMR. Its principal contribution lies in the evaluation of myocardial ischemia, coronary microvascular dysfunction, and abnormalities of myocardial metabolism and autonomic innervation. These are pathophysiologic processes that play a central role in symptom development, disease progression, and arrhythmic risk but are not reliably inferred from wall thickness alone [96].

Myocardial perfusion imaging with single-photon emission computed tomography (SPECT) commonly reveals fixed or reversible perfusion defects in patients with HCM, frequently localized to hypertrophied myocardial segments. These abnormalities reflect a combination of replacement fibrosis and stress-induced ischemia rather than obstructive epicardial coronary disease and therefore lack specificity for identifying flow-limiting coronary stenoses [96]. Positron emission tomography (PET), by contrast, enables absolute quantification of myocardial blood flow and coronary flow reserve and has consistently demonstrated a markedly attenuated vasodilator response in HCM, even in the presence of angiographically normal coronary arteries [97,98]. Impaired stress myocardial blood flow measured by PET has been associated with adverse clinical outcomes, including progression to heart failure and increased cardiovascular mortality [96,97].

Notably, PET-derived quantification of myocardial blood flow and coronary flow reserve provides a robust, noninvasive assessment of microvascular function, enabling early detection of ischemia even before structural changes become evident. Reduced coronary flow reserve has emerged as a key marker of disease severity and has been consistently associated with adverse outcomes, including progression to advanced heart failure and increased risk of cardiovascular events. These metrics offer incremental prognostic value beyond conventional imaging, supporting their role in refining risk stratification and identifying high-risk HCM phenotypes [99].

Coronary microvascular dysfunction in HCM arises from a complex interplay between intravascular and extravascular mechanisms. Structural remodeling of small intramural coronary arterioles and a relative reduction in capillary density limit maximal flow capacity, while extravascular compressive forces related to increased myocardial mass, elevated intracavitary pressures, diastolic dysfunction, and dynamic outflow tract obstruction further impair perfusion, particularly in the subendocardial layers during stress [98,100]. Quantitative and transmural perfusion analyses have demonstrated reversal of the normal endocardial-to-epicardial flow gradient under hyperemic conditions, underscoring the vulnerability of the subendocardium to ischemia in this disease [98,100].

Beyond perfusion, nuclear techniques provide insight into myocardial metabolism and autonomic function. Altered myocardial substrate utilization may precede overt fibrosis or perfusion abnormalities, and imaging of cardiac sympathetic innervation has been linked to ventricular arrhythmias and adverse outcomes [96]. Collectively, these findings position nuclear imaging as a valuable adjunct for phenotyping and risk assessment in selected patients with hypertrophic cardiomyopathy.

## 6. Biomarker-Based Assessment of Disease Burden in HCM

In clinical practice, only a limited number of circulating biomarkers are routinely used in hypertrophic cardiomyopathy, primarily for risk stratification and monitoring. These include natriuretic peptides and cardiac troponins. Other biomarkers remain investigational and are not yet incorporated into routine management.

### 6.1 Established Biomarkers (BNP, Troponin)

The diagnosis of HCM is based on clinical, imaging, and sometimes genetic findings, but since prognostication remains limited except in patients with high-risk indicators for sudden cardiac death, biomarkers that provide additional prognostic information could be useful for assessing the risk of arrhythmia, heart failure, sudden cardiac death, and the effectiveness of pharmacologic or interventional therapies such as surgical myectomy and alcohol septal ablation [101].

Biomarkers of myocardial wall stress include natriuretic peptides (NPs) such as brain natriuretic peptide (BNP), atrial natriuretic peptide (ANP), and N-terminal pro–B-type natriuretic peptide (NT-proBNP) [102]. Their precursor, proBNP, is secreted by ventricular myocytes in response to increased wall stress and is cleaved by corin into the active hormone BNP and the inactive fragment NT-proBNP [103].

In HCM, BNP levels are markedly elevated compared with controls, with higher concentrations observed in obstructive than in non-obstructive cases [104,105]. Both BNP and ANP show a positive correlation with the left intraventricular pressure gradient, reflecting the degree of obstruction [104,105].

High-sensitivity cardiac troponins T and I are highly sensitive and specific markers that can detect even minimal cardiomyocyte injury, extending their use beyond myocardial infarction [106]. Elevated troponin in HCM may result from relative myocardial ischemia caused by hypertrophied myocardium outgrowing its blood supply or from direct myocyte damage due to underlying genetic mutations [107].

Both natriuretic peptides and troponins are associated with adverse clinical outcomes, including heart failure, arrhythmias, and mortality, and are recommended for prognostic assessment in current guidelines [108]. Patients with overt HCM exhibit higher levels of these biomarkers compared with subclinical mutation carriers, supporting their role in tracking disease expression [109]. Exercise further increases BNP and troponin levels, particularly in patients with silent ischemia, suggesting that integration with stress testing may enhance risk stratification [109,110,111].

In addition, both biomarkers are associated with myocardial fibrosis, with troponin demonstrating particular utility in identifying patients with extensive fibrosis who may benefit from further evaluation with CMR imaging [112,113,114]. Troponin elevation has also been linked to disease progression, including impaired systolic function and transition toward end-stage dilated HCM [115,116,117].

Importantly, the combined assessment of natriuretic peptides and troponins improves prognostic stratification compared with either biomarker alone, highlighting their complementary clinical roles [118].

Both natriuretic peptides and troponins are associated with adverse clinical outcomes, including heart failure, arrhythmias, and mortality, and are recommended for prognostic assessment in current guidelines [108]. Patients with overt HCM exhibit higher levels of these biomarkers compared with subclinical mutation carriers, supporting their role in tracking disease expression [109]. Exercise further increases BNP and troponin levels, particularly in patients with silent ischemia, suggesting that integration with stress testing may enhance risk stratification [109,110,111].

In addition, both biomarkers are associated with myocardial fibrosis, with troponin demonstrating particular utility in identifying patients with extensive fibrosis who may benefit from further evaluation with CMR imaging [112,113,114]. Troponin elevation has also been linked to disease progression, including impaired systolic function and transition toward end-stage dilated HCM [115,116,117].

Importantly, the combined assessment of natriuretic peptides and troponins improves prognostic stratification compared with either biomarker alone, highlighting their complementary clinical roles [118].

In HCM patients treated with myosin inhibitors, NT-proBNP and high-sensitivity cardiac troponin I (hs-cTnI) consistently decrease, reflecting reductions in myocardial stress and subclinical injury [119]. Dose-dependent reductions in NT-proBNP and hs-cTnI were observed with use of *Aficamten*, accompanied by improvements in New York Heart Association (NYHA) functional class [120]. Similarly, treatment with *Mavacamten* led to decreases in NT-proBNP and hs-cTnI, which correlated with improved diastolic function, lower left atrial volume index, and reduced left ventricular mass index [121]. These results highlight NT-proBNP and hs-cTnI as established biomarkers for monitoring therapeutic response to myosin inhibitors, serving as early indicators of improved myocardial function and decreased wall stress.

### 6.2 Emerging Biomarkers

Several circulating biomarkers have been explored to better characterize the biological processes underlying HCM, particularly inflammation and fibrosis. However, their clinical utility remains unproven, and none are currently recommended for routine use.

Markers of inflammation and apoptosis, including interleukin-6, tumor necrosis factor-α (TNF-α), and soluble Fas ligand, are elevated in HCM and may contribute to disease progression [122]. TNF-α levels are particularly increased in advanced stages, supporting a role for inflammatory and apoptotic pathways in adverse remodeling [123].

Given the central role of myocardial fibrosis in HCM, biomarkers of extracellular matrix turnover have also been investigated. These include procollagen types I and III, matrix metalloproteinases, and their tissue inhibitors [124], as well as proteins such as galectin-3, growth differentiation factor-15, and soluble suppression of tumorigenesis 2 [125,126]. Among these, galectin-3 has been most extensively studied; although it correlates with disease severity in some cohorts, it does not appear to closely reflect the magnitude of myocardial hypertrophy in HCM [109,127].

Overall, while these biomarkers provide insight into key disease mechanisms, their incremental value beyond established markers remains uncertain.

### 6.3 Experimental Biomarkers

Several novel biomarkers remain at an early stage of investigation and are currently limited to small or exploratory studies.

Autoantibodies targeting G-protein coupled and muscarinic receptors have been reported in HCM, with higher levels observed in patients with a family history of sudden cardiac death or atrial fibrillation [128]. Antibodies against calreticulin have also been identified, although their clinical significance remains unclear [129].

Non-coding RNAs, including microRNAs and circular RNAs, are involved in the regulation of myocardial hypertrophy and fibrosis. Circulating miR-29a has been associated with both processes, and combined microRNA profiling may improve detection of diffuse fibrosis. Similarly, certain circular RNAs have been linked to the severity of left ventricular outflow tract obstruction [130].

Endothelial and hemostatic alterations have also been described. Endothelin-1 and its precursor are elevated and associated with myocardial fibrosis and adverse outcomes [131]. In addition, increased shear stress in obstructive HCM may lead to degradation of von Willebrand factor multimers, contributing to bleeding risk and correlating with outflow tract gradients; these abnormalities may improve following septal reduction therapies [132].

## 7. Genetic Testing

Genetic testing is recommended for all patients with a clinical diagnosis of HCM to confirm the diagnosis, identify the molecular etiology, and facilitate cascade screening of at-risk relatives [108], as schematically illustrated in Fig. 6. Hence, genetic testing should be performed in the proband using targeted disease-specific panels that include the eight core sarcomere genes with the strongest evidence of causality: *MYBPC3* (myosin-binding protein C3), *MYH7* (β-myosin heavy chain), *TNNT2* (troponin T2), *TNNI3* (troponin I3), *TPM1* (α-tropomyosin), *MYL2* (myosin regulatory light chain 2), *MYL3* (myosin essential light chain 3), and *ACTC1* (α-actin). These genes account for over 90% of genotype-positive HCM cases, with *MYBPC3* and *MYH7* representing approximately 45% and 25% of sarcomeric HCM, respectively [133]. Phenocopies (genes associated with HCM-like syndromes) should be included in first-tier genetic testing if there are clinical manifestations suggestive of syndromic or metabolic disorders, especially those conditions with available specific treatments such as Anderson-Fabry disease (*GLA*), Danon disease (*LAMP2*), *PRKAG2* syndrome, or familial amyloidosis (*TTR*) [134]. Expanding to larger panels generally does not add diagnostic value and may increase inconclusive findings. However, compared to targeted panels, many studies have investigated utilizing whole exome sequencing (WES) and whole genome sequencing (WGS) to improve diagnostic yield in cases with prior inconclusive genetic testing [135].

**Fig. 6.**
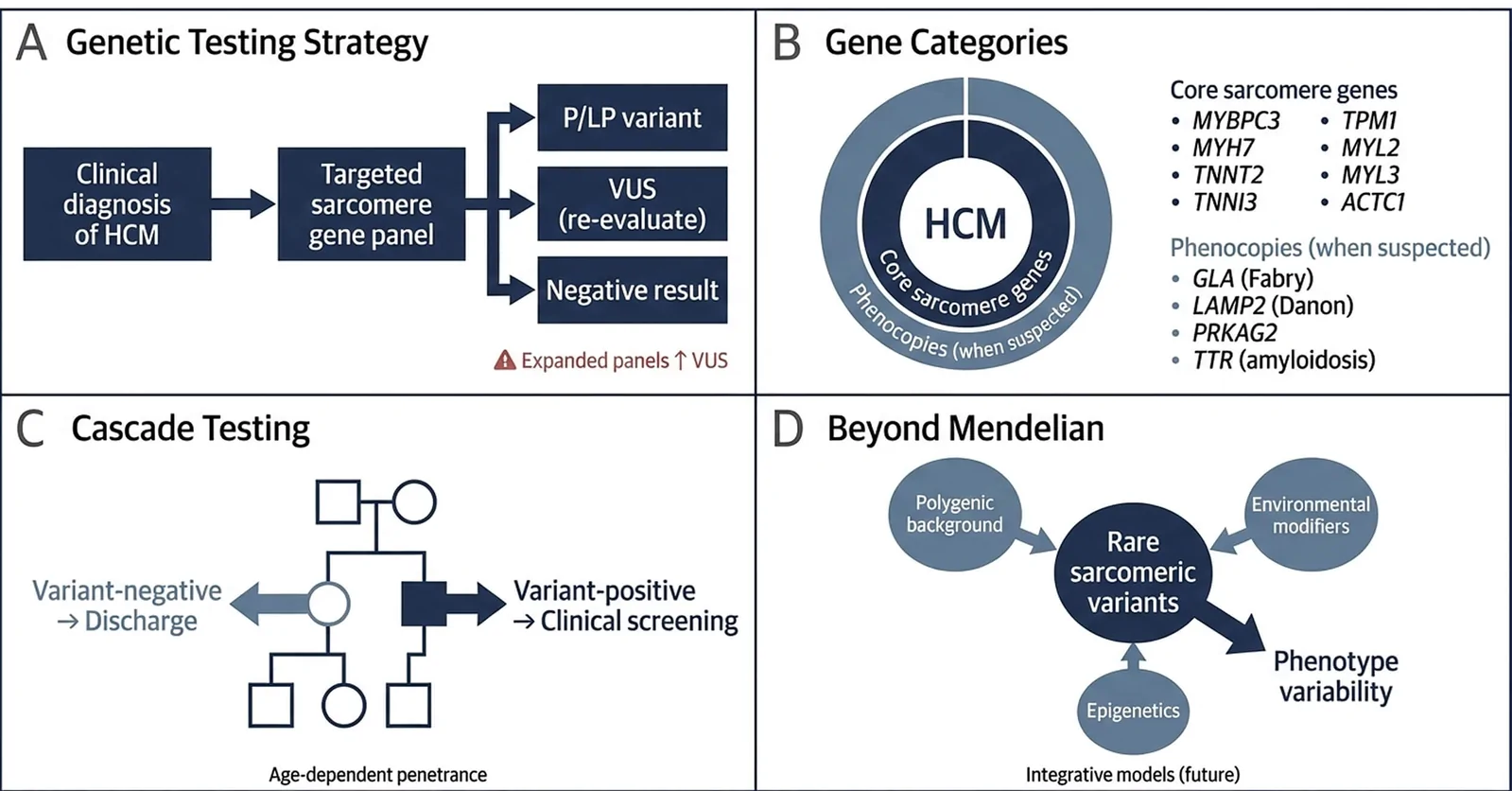
**Genetic architecture in hypertrophic cardiomyopathy**. Schematic overview of contemporary genetic testing strategies (A), core sarcomere and phenocopy genes (B), cascade family screening (C), and the multifactorial influences that modulate phenotypic expression in HCM (D). P/LP, pathogenic or likely pathogenic; VUS, variants of uncertain significance. Created in BioRender.

Pathogenic or likely pathogenic (P/LP) variants in genes encoding sarcomere proteins are identified in about 30–40% of all HCM cases, with higher yields in familial disease when compared to the general population [136]. Variant interpretation and clinical application of genetic results require standardized frameworks and expert supervision. Variants of uncertain significance (VUS) are common in HCM, particularly with expanded panels and exome sequencing, and should not be used to guide clinical management or determine family screening [137]. Periodic re-evaluation of VUS is essential as new evidence emerges. Importantly, genotype–phenotype correlations extend beyond penetrance alone; specific sarcomere mutations have been associated with differences in age of onset, degree of hypertrophy, heart failure progression, and arrhythmic risk, although these associations are probabilistic and should not be used in isolation for risk stratification [137].

Focused cascade genetic testing is recommended for phenotype-negative first-degree relatives when a variant with decisive evidence of pathogenicity has been identified in the proband. This recommendation applies regardless of age, as recent data show that a significant percentage of HCM patients can manifest before 10 years of age with high morbidity and mortality [108,136]. Relatives who do not carry the familial P/LP variant can be reassured and discharged from further follow-up, as their risk of developing HCM is similar to that of the general population. Conversely, relatives who carry a P/LP variant are at risk for developing HCM and should undergo interval clinical screening to detect the emergence of clinically overt disease [30,136]. The frequency of screening depends on age, annually during adolescence and early adulthood, and every 3–5 years later in adulthood. Interestingly, penetrance varies substantially by gene, with family-based studies showing penetrance of approximately 32% for *MYL3*, 55% for *MYBPC3*, 60% for *TNNT2* and *TNNI3*, and 65% for *MYH7*. In contrast, population-based genetic studies demonstrated that P/LP sarcomere variants are present in the background population at a low prevalence [136].

These observations underscore that HCM phenotype results from multifactorial influences that extend beyond simple Mendelian inheritance patterns [136]. Accumulating evidence suggests that many cases follow a complex polygenic inheritance model, where multiple genetic variants interact with environmental factors and epigenetic modifications to influence disease expression and phenotypic presentation [2]. Previous studies of familial and non-familial HCM patients demonstrated that hypertension and obesity were more prevalent in non-familial HCM cases [2]. Comprehensive patient evaluation should therefore include assessment of modifiable environmental risk factors alongside genetic testing, as lifestyle interventions targeting obesity, hypertension control, and appropriate exercise restriction may influence disease trajectory and outcomes. Future directions include refinement of polygenic risk scores and integrative models combining genetics with imaging and clinical variables, although these approaches remain investigational and are not yet ready for routine clinical use [138].

## 8. Artificial Intelligence (AI) and Machine Learning Tools

AI is rapidly transforming cardiovascular diagnostics, offering opportunities to detect HCM earlier and with greater accuracy. Machine learning approaches, particularly deep learning models, can be trained to recognize subtle cardiac imaging and ECG abnormalities that often precede clinically recognizable disease. AI enabled tools have the potential to expand early recognition, improve referral pathways, and improve population level screening.

### 8.1 Overview of AI Models Used in HCM Diagnosis

AI systems use computational models to perform cognitive and interpretive tasks that had previously relied on human expertise [139]. These systems are well suited for HCM where subtle early disease manifestations contribute to underdiagnosis in a potentially fatal disease [5,6,140]. AI-based models boast promise for their ability to detect subtle and even early patterns of disease in ECG, echo, and CMR [141].

Machine learning (ML) which uses predictive relationship models and a sub-type of machine learning called deep learning have shown promise when applied to HCM [140]. These models can be applied to structural clinical variables and ECG derived measurements to discriminate patients with HCM from healthy or disease-mimicking populations and identify early subtle manifestations of HCM [141,142].

Convolutional Neural Networks (CNNs) are a type of DL model designed to process structured grid data, such as ECGs and images. CNNs have been used to identify regional hypertrophy [143], myocardial disarray signatures [144], along with extracting morphological features such as QRS fragmentation and repolarization changes without manual feature engineering [142]. CNNs have shown some of the highest reported AUROC values in HCM AI diagnostics [145,146], as shown in Fig. 7 (Ref. [6]).

**Fig. 7.**
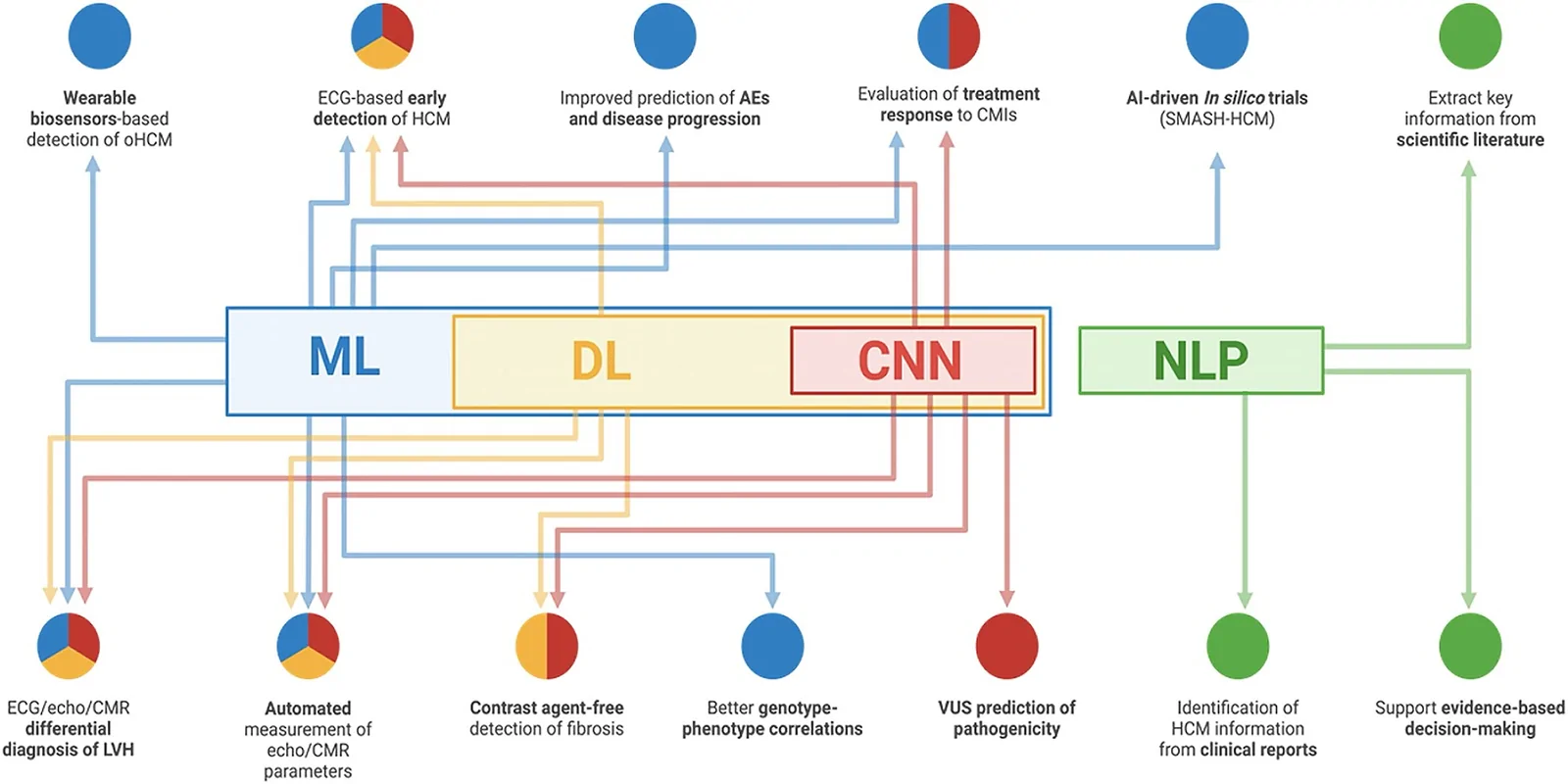
**Diagram illustrating AI taxonomy and the different applications in HCM**. The figure illustrates the hierarchy of AI models, with machine learning (ML) encompassing DL, which includes Convolutional Neural Networks (CNNs), and natural language processing (NLP) as a separate approach [6]. Created in BioRender.

Lastly, natural language processing (NLP) models can mine text to extract relevant information and have been used to generate personalized risk profiles. These can consider clinical notes, family history, and patient narratives to enrich risk prediction models [147].

### 8.2 Detection of HCM Using ECG

Initial approaches to harnessing AI in screening and diagnosis of HCM have focused on ECG. ECG was previously limited in its screening ability because it has relied on manual or semi-automated detection of ECG findings, such as LVH, left axis deviation, prominent Q waves, and T-wave inversions. Such approaches had insufficient sensitivity and specificity to justify routine ECG screening for HCM [148]. AI however has allowed for an evolution in ECG evaluation of HCM and has dramatically increased the diagnostic and screening potential of this study.

Initial studies that looked at CNN models trained to detect HCM on ECG demonstrated that after training the model could identify patients with HCM with high diagnostic accuracy (area under the curve (AUC) of 0.96) [141]. Furthermore, in the subset of patients with HCM with “normal” ECGs by conventional criteria the model still held this level of diagnostic accuracy demonstrating the capacity to detect latent or subclinical disease [141]. Recent meta-analyses have continually demonstrated strong performance across larger more diverse populations continuing to support their role as a screening or case-finding tool rather than a standalone diagnostic test [145,146].

Importantly, real-world clinical implementation studies now show that AI-ECG tools can uncover previously unrecognized HCM. In these studies, the models flagged ECGs suspicious for HCM. This prompted follow up evaluation and led to several new diagnoses of HCM. These trials not only increased the accuracy of diagnosis but improved time to diagnosis and treatment, compared to previous diagnostic pathways [149,150].

An additional consideration is the increasing use of cardiac myosin inhibitors in the treatment of HCM. These medications reduce left ventricular mass and repolarization abnormalities over time [151]. Because these models are trained on untreated phenotypically overt HCM, ECG changes caused by therapy may limit these models’ accuracy.

Despite this high accuracy in diagnosis, limitations remain. Considerable heterogeneity exists across the datasets used, which could make broad adoption a challenge [145,146]. The positive predictive value (PPV) in screening settings, especially in low-prevalence populations, may be modest despite high specificity. Some models may also suffer from dataset bias (e.g., overrepresentation of certain demographics or clinical centers), raising questions about generalizability in diverse or under-represented populations. Thus, while AI-ECG is unlikely to supplant imaging-based diagnosis, it shows strong promise as a first-line screening tool or “gatekeeper” to triage patients toward confirmatory echocardiography or CMR.

### 8.3 AI and Echocardiography

AI models applied to echocardiography have emerged as a promising tool in assisting clinicians in identifying HCM. One challenge in clinical practice is distinguishing HCM from other causes of LVH. Early studies demonstrated that AI-assisted echocardiography could automate beat-to-beat measurements of ventricular wall thickness, internal diameters, and other key parameters with accuracy comparable to human operators but at far greater speed and consistency [143]. More importantly, these models moved beyond measurement and assisted with disease classification, helping to differentiate HCM from other causes of ventricular hypertrophy and prompting further evaluation such as CMR imaging or genetic testing when appropriate [143]. In this way, AI has the potential to function as a decision-support tool that helps clinicians move from identifying hypertrophy to determining its underlying cause.

Point-of-care ultrasound (POCUS) has grown rapidly and recent studies have looked at applying AI models to this to improve early detection of HCM and improve accessibility of testing. AI models trained on apical four-chamber views have been able to identify patients with HCM and other cardiomyopathies and, in some cases, flagged abnormalities years before a formal clinical diagnosis was made [150]. This raises the possibility that AI-enabled ultrasound could be used for opportunistic screening in a variety of settings, including primary care, emergency departments, and community screening programs, potentially identifying patients earlier in the disease course.

Despite these promising applications, there are several limitations before widespread clinical adoption is feasible. Many models have been trained on high-quality echo data from single academic centers, and it would be expected that performance will be reduced in community settings where image quality, equipment, and operator experience vary. These models also rely on patterns that are not easily interpretable in current echocardiographic measurements, which makes insight into the AI’s decision-making process to flag a given study making interpretability challenging. This could also become a challenge when going to regulate the technology.

### 8.4 AI and Deep Learning in CMR

The integration of AI into CMR imaging is rapidly advancing across all aspects of the clinical workflow, from image acquisition through prognostic risk stratification, with several applications already approaching clinical implementation.

### 8.5 Automated Segmentation and Quantification

Deep learning algorithms, particularly Convolutional Neural Networks, have achieved human-level accuracy in segmenting the LV and right ventricle (RV) demonstrating Dice similarity coefficients exceeding 0.90 [152,153,154]. This automates the calculation of mass and volumes, reducing processing time from approximately 20 minutes to seconds and eliminating inter-observer variability [153].

### 8.6 Virtual Native Enhancement (VNE)

VNE technology generates LGE-equivalent images without gadolinium administration by combining cine imaging and native T1 maps through generative adversarial networks [155,156]. In patients with myocardial infarction, VNE demonstrated a strong correlation with conventional LGE for scar quantification (R = 0.89, intraclass correlation coefficient = 0.94) and transmural assessment (R = 0.84, intraclass correlation coefficient = 0.90) [155]. Notably, VNE provided superior image quality compared to conventional LGE while maintaining high diagnostic accuracy (84% overall accuracy, 100% specificity, 77% sensitivity) [155]. Similar performance has been validated in HCM, where VNE achieved high visuospatial agreement with LGE for both hyperintensity (r = 0.77–0.79) and intermediate-intensity lesions (r = 0.70–0.76) [156]. This technology offers particular benefit for patients with renal insufficiency or gadolinium allergies [157].

### 8.7 Prognostic AI

Machine learning models demonstrate superior performance compared to traditional clinical risk calculators for predicting SCD and major adverse cardiovascular events [158,159]. Deep learning approaches can identify radiomic features, texture patterns invisible to human observers, directly from raw CMR data [160,161]. Machine learning models in HCM have achieved AUCs of 0.830 for cardiovascular event prediction, significantly exceeding Cox regression (0.717) and HCM Risk-SCD models (0.720) [161]. These AI-derived risk scores integrate multiple data streams including patient demographics, clinical variables, and quantitative imaging features to generate nonlinear predictive models [162].

The American Heart Association notes that AI applications in CMR extend to myocardial tissue characterization, prediction of SCD risk, and guidance of ablation procedures by analyzing fibrosis patterns [158]. However, challenges remain for widespread clinical implementation, including the need for standardized protocols, external validation in diverse populations, and regulatory approval.

### 8.8 Future Directions

Diagnosis and risk assessment in HCM may be strengthened by a cohesive multimodality approach in which echocardiography remains foundational but is complemented by advanced imaging, electrophysiologic assessment, and genetics. Greater standardization of execution and reporting across modalities, including echocardiographic measures of diastolic function and obstruction, quantitative fibrosis by CMR, parametric mapping indices, and perfusion metrics from stress imaging, would improve comparability across centers and support more consistent clinical practice. Aligning echocardiographic findings with downstream testing will be essential for a coherent diagnostic approach.

Longitudinal evaluation represents another critical area for advancement. Serial echocardiographic evaluation of remodeling, obstruction, and atrial size, when combined with temporal changes in ECG features, myocardial fibrosis burden, and perfusion defects, may better capture disease evolution than single time-point assessments. Such an approach could enable more individualized surveillance and improve timing of therapeutic interventions. Artificial intelligence offers a promising framework for integrating data from different diagnostic modalities into a unified diagnostic and prognostic model. However, meaningful clinical adoption will require robust external validation and careful integration into clinical workflow that augments, rather than replaces, clinical judgment.

## 9. Conclusion

Non-echocardiographic modalities provide essential biological and prognostic information beyond the detection of hypertrophy. ECG, CT, CMR, nuclear imaging and genetic testing all provide complementary insights into electrical remodeling, tissue substrates, perfusion and microvascular disease. They also provide invaluable insight to molecular causality which leads to enhanced phenotyping, risk stratification and familial screening. Integrating echocardiographic and non-echocardiographic modalities, perhaps best combined with the use of validated AI systems, offers a more clinically meaningful approach for the diagnosis and longitudinal management of HCM.

In clinical practice, multimodality findings in HCM should directly inform management decisions rather than serve as isolated diagnostic observations. If extensive LGE (≥15%) or an apical aneurysm is identified on CMR, then intensified sudden cardiac death risk assessment and consideration of prophylactic ICD implantation may be warranted, particularly in the presence of additional risk markers. If stress perfusion imaging demonstrates severe microvascular dysfunction or reduced myocardial perfusion reserve, then closer surveillance for heart failure progression and adverse remodeling should be considered. Furthermore, if low QRS voltage appears disproportionate to wall thickness, clinicians should evaluate for phenocopies such as cardiac amyloidosis rather than assuming sarcomeric HCM alone. If genotype-positive/phenotype-negative status is identified, then periodic imaging surveillance and cascade family screening remain important despite the absence of overt hypertrophy. If biomarkers such as NT-proBNP or hs-cTnI remain persistently elevated, then further assessment for fibrosis burden, worsening obstruction, or advanced disease progression may be indicated. Finally, if AI-assisted ECG or imaging tools identify high-risk or subclinical patterns, in that case confirmatory multimodality imaging and expert clinical evaluation should precede management decisions.

### Limitations

This review is limited by its narrative design and by its reliance on data availability across various research databases, which is subject to selection bias. Heterogeneity in populations, imaging protocols, and biomarker cut-offs limits comparability between studies. Furthermore, the state-of-the-art techniques of advanced nuclear imaging and AI-based prognostic models have not all been validated, nor has their value been confirmed in diverse real-world settings.
